# Knowledge, Attitude, Benefits, Risks, Barriers, Professional Impact, and Preparedness of Nursing Students Toward the Utilization of Artificial Intelligence in Healthcare

**DOI:** 10.3390/nursrep16050154

**Published:** 2026-05-04

**Authors:** Awatif Alrasheeday, Aeshah Abdulaziz ALhawsawi, Bushra Alshammari, Sameer A. Alkubati, Wiem Aouicha, Mohamed Ayoub Tlili, Abdulhafith Alharbi, Bahia Galal Siam, Soha Mahmoud, Badria Elamin, Layla Alshammari, Hajer I. Motakef, Tahani Alkhammali, Ahad Alanazi, Fatimah Alshammari, Huda Alshammari, Ruqayyah Abdullah Almohammed, Ruba Abdulaziz Alomran

**Affiliations:** 1 Nursing Administration Department, College of Nursing, University of Hail, Ha’il 2440, Saudi Arabia; w.aouicha@uoh.edu.sa (W.A.); mo.tlili@uoh.edu.sa (M.A.T.); 2 King Salman bin Abdulaziz Medical City, Al Madinah Al Munawwarah 42319, Saudi Arabia; ooshoo_00@hotmail.com; 3 Medical Surgical Nursing Department, College of Nursing, University of Hail, Ha’il 2440, Saudi Arabia; Bu.alshammari@uoh.edu.sa (B.A.); s.alkubati@uoh.edu.sa (S.A.A.); b.siam@uoh.edu.sa (B.G.S.); b.alamin@uoh.edu.sa (B.E.); lay.alshammari@uoh.edu.sa (L.A.); 4 Faculty of Medicine of Sousse, University of Sousse, Sousse 4003, Tunisia; 5 Department of Psychiatric and Mental Health Nursing, College of Nursing, University of Hail, Ha’il 2440, Saudi Arabia; ay.alharbi@uoh.edu.sa; 6 Community Health Nursing Department, College of Nursing, University of Hail, Ha’il 2440, Saudi Arabia; s.mahmoud@uoh.edu.sa; 7 Department of Maternal and Child Health, College of Nursing, University of Hail, Ha’il 2440, Saudi Arabia; ha.matekef@uoh.edu.sa (H.I.M.); t.alkhammali@uoh.edu.sa (T.A.); 8 Emergency Department, King Salman Specialist Hospital, Hail Health Cluster, Ha’il 2440, Saudi Arabia; aalanazi712@moh.gov.sa (A.A.); falshammari125@moh.gov.sa (F.A.); halshammari63@moh.gov.sa (H.A.); rualmohammed@moh.gov.sa (R.A.A.); raalomran@moh.gov.sa (R.A.A.)

**Keywords:** artificial intelligence, nursing, nursing students, knowledge, attitudes, perceived benefits, perceived risks, barriers, preparedness, healthcare technology, nursing education

## Abstract

**Background/Objectives**: Artificial intelligence (AI) is increasingly used in healthcare to support clinical decision-making, patient monitoring, and administrative tasks. Nurses are expected to work with these technologies. However, the evidence suggests that their knowledge and preparedness remain limited. As future healthcare providers, nursing students must be prepared to integrate AI into their practice. This study aimed to assess nursing students’ knowledge, attitudes, perceived benefits and risks, barriers, professional impact, and preparedness toward AI in healthcare. **Methods**: This cross-sectional descriptive study was conducted between April and July 2024 at the College of Nursing, University of Hail, Saudi Arabia. A convenience sample of 320 undergraduate nursing students completed an online structured questionnaire that assessed their demographics, knowledge, attitudes, perceived barriers, benefits, risks, professional impact, and preparedness. Data were analyzed using IBM SPSS version 27 with descriptive statistics. Inferential analyses, including independent *t*-tests and one-way ANOVA, were performed to examine differences between groups. Pearson’s correlation was used to identify correlations between the study variables. Statistical significance was set at *p* < 0.05. **Results**: Most students (79.7%) had poor AI knowledge, whereas 52.5% reported positive attitudes. Older students (≥24 years) and internship students showed significantly more positive attitudes (*p* < 0.001). Knowledge was weakly correlated with attitudes (r = 0.147), benefits (r = 0.222), and risks (r = 0.152). Attitudes were weakly positively correlated with benefits (r = 0.243) and negatively correlated with barriers (r = −0.219). **Conclusions**: Despite their positive attitudes, nursing students showed limited knowledge and preparedness. Integrating AI education and practical training into nursing curricula is therefore essential. These findings should be interpreted cautiously given the cross-sectional design, single-institution sampling, and reliance on self-reported measures, which may limit generalizability.

## 1. Introduction

Artificial intelligence (AI) has rapidly emerged as a transformative force across many sectors, particularly in healthcare, where it is increasingly used to enhance clinical decision-making, improve diagnostic accuracy, optimize workflow, and support patient monitoring [1,2]. In the context of nursing practice, AI is not a monolithic entity but encompasses a spectrum of technologies. These range from machine learning algorithms embedded in electronic health records to predict patient deterioration (e.g., risk of sepsis or falls) and support diagnostic imaging to generative AI tools that assist with clinical documentation, summarizing patient data, and power patient-facing chatbots for education and monitoring [3]. Applications of AI now extend from predictive analytics and clinical decision support systems to robotic assistance and personalized medicine, offering significant opportunities to improve the quality of care and reduce human error [4]. As healthcare systems continue to digitalize, the integration of AI is expected to accelerate and reshape professional roles and clinical practices. Nurses play a central role in patient care and are among the primary end users of AI-based healthcare technologies [5]. AI tools are already used in nursing practice to predict patient deterioration, manage complex data, and automate routine tasks, allowing nurses to focus on direct patient care [6,7]. However, the successful implementation of AI depends not only on technological readiness but also on the knowledge, attitudes, and preparedness of healthcare professionals who use these systems [7,8]. Despite growing interest in AI, evidence suggests a gap between its potential and readiness for future healthcare providers. Several studies have reported that although healthcare students and professionals generally express positive attitudes toward AI, they often demonstrate limited knowledge, technical understanding, and practical experience [9,10,11]. This mismatch may hinder effective adoption and create resistance or ethical concerns related to data privacy, accountability, and job displacement [12]. Among nursing students, exposure to AI remains inconsistent and largely theoretical. Many nursing curricula do not yet include structured AI education or hands-on training, which may contribute to uncertainty and low confidence in using AI technologies [13,14]. While some researchers argue that AI will enhance nursing roles and improve patient outcomes, others caution that overreliance on automated systems may reduce critical thinking and threaten professional autonomy [12,15,16].

This study is guided by the Technology Acceptance Model (TAM), which provides a theoretical framework for understanding individuals’ adoption of new technologies. TAM is a well-established theoretical framework that posits perceived usefulness and perceived ease of use as primary determinants of users’ behavioral intention to adopt a new technology [7,17]. In the context of AI in nursing, this model offers a valuable lens for understanding how students’ perceptions shape their readiness to use these tools. Specifically, students’ knowledge of AI and their perceptions of its benefits directly inform their judgment on its perceived usefulness in patient care. Conversely, perceived risks, ethical concerns, and educational barriers can negatively impact the perceived ease of use and overall usefulness, thereby hindering acceptance [12,18]. Within this framework, “preparedness” is conceptualized as an outcome closely linked to the intention to use AI, reflecting students’ confidence in their ability to apply technology in practice. Accordingly, the present study operationalizes TAM by examining the relationships between these constructs, thereby enabling a theory-driven investigation of the factors influencing nursing students’ readiness to engage with AI technologies.

Recent research in this region has begun to map nursing students’ perceptions of AI, often revealing a landscape of high enthusiasm and limited understanding [9]. However, a more complex picture is emerging, sometimes described as a “trust–optimism paradox,” where positive attitudes coexist with significant skepticism toward the reliability of AI-generated clinical recommendations [19]. This study advances beyond these descriptive findings by employing a multidimensional approach to investigate the interplay between Saudi nursing students’ knowledge, attitudes, and crucially, their perceived professional impact and clinical preparedness. While prior work has identified knowledge gaps, our study specifically examined the disconnect between theoretical positivity and practical readiness, a critical factor influencing safe and ethical AI adoption in future practice [20]. By analyzing the correlations among different key constructs, including barriers, risks, and professional impact, this study seeks to provide a more nuanced understanding of the factors that either facilitate or hinder the transition from positive attitudes to competent real-world utilization of AI in nursing care. This systemic insight is essential for moving beyond generic calls for “more education” toward the development of targeted, competency-based curricula that address specific gaps in students’ preparedness [3].

Therefore, this study aimed to assess nursing students’ knowledge, attitudes, perceived benefits and risks, barriers, professional impact, and preparedness toward the utilization of AI in healthcare. By identifying gaps and relationships among these factors, this study provides evidence to support the integration of AI-focused education into nursing curricula and promote the safe and effective adoption of AI in clinical practice.

## 2. Materials and Methods

### 2.1. Study Design

A cross-sectional descriptive study design was employed to assess nursing students’ knowledge, attitudes, perceived benefits and risks, barriers, professional impact, and preparedness toward the utilization of artificial intelligence (AI) in healthcare.

### 2.2. Setting

This study was conducted at the College of Nursing, University of Ha’ il, located in the northern region of Saudi Arabia. The college offers undergraduate nursing education through a structured curriculum that includes theoretical courses and clinical training. The participants were nursing students enrolled in an undergraduate programme during the study period. The college enrolls approximately 1000 undergraduate students across different academic levels, including general nursing and midwifery programmes.

### 2.3. Study Population and Sampling

The study population consisted of undergraduate nursing students enrolled at the University of Ha’ il during the academic years 2023–2024. Convenience sampling was used to recruit participants who were willing to participate. The required sample size was calculated using G*Power version 3.1.9.4. An a priori power analysis was conducted for a bivariate correlation test (exact test; bivariate normal model) to determine the minimum sample size required to detect a statistically significant correlation. The analysis assumed an anticipated effect size (correlation coefficient) of r = 0.20, representing a small effect according to Cohen’s guidelines. A one-tailed test was specified with a significance level (α) of 0.05 and statistical power of 0.95. Based on these parameters, the analysis indicated that a minimum sample size of 266 participants is required to achieve an actual power of 0.950 to detect the expected correlation effect. To improve the representation and account for likely non-responders, we distributed all students. A total of 320 completed surveys were received and included in the final analysis, with a response rate of 32.0%.

### 2.4. Data Collection

Data were collected between April and July 2024, using an online self-administered questionnaire created using Google Forms. The survey link was distributed via university email to all the nursing students. A reminder email was sent two weeks after the initial distribution to improve the response rate. The questionnaire was configured such that all items were mandatory, requiring participants to respond to each question before proceeding to the next question. Consequently, no missing data were present in the dataset and only fully completed responses were included in the analysis. Participation was voluntary and anonymous.

### 2.5. Instrument

The structured questionnaire consisted of four sections:

Demographic and Academic Characteristics: age, gender, residence, and academic year.

Knowledge, Attitudes, and Perceived Barriers: Adapted from Al-Qerem et al. (2023) [21], seven knowledge items (yes/no), 10 attitude items (5-point Likert scale), and seven barrier items were used.

Seven items assessed participants’ basic understanding of AI concepts and applications. Responses were dichotomous (yes/no), with each correct or affirmative response scored as 1 and each negative response scored as 0. The total knowledge score ranged from 0 to 7, with higher scores indicating greater knowledge of AI. To facilitate interpretation, knowledge was categorized into three levels based on the percentage of the total score. Scores below 60% of the total possible score were classified as poor knowledge, scores between 60% and 75% were categorized as fair knowledge, and scores ≥80% were considered good knowledge [22].

Attitude scores were classified as negative (<60%) or positive (≥60%), based on the percentage of the total possible score.

Perceived Benefits and Risks: Adapted from Perrier et al. (2022) [15], including nine benefit and five risk items rated as yes, no, or neutral.

Professional Impact and Preparedness: assessed using the Shinners Artificial Intelligence Perception Survey, including six professional impact and four preparedness items (5-point Likert scale) [16].

The measurement instruments used in this study were adapted from the previously validated scales [15,16,21]. A pilot test was conducted with 30 nursing students to ensure comprehensibility of the items among the participants. The internal consistency reliability of the instruments in the current study was assessed using Cronbach’s alpha. The results demonstrated acceptable reliability for all subscales, with Cronbach’s alpha values of (α = 0.712) for the knowledge scale, (α = 0.842) for the attitude scale, (α = 0.756) for the benefits scale, (α = 0.752) for the risk scale, (α = 0.814) for the barrier scale, (α = 0.850) for the professional impact scale, and (α = 0.700) for the preparedness scale, indicating satisfactory internal consistency.

### 2.6. Data Analysis

Data were analyzed using IBM SPSS Statistics version 27 (IBM Corp., Armonk, NY, USA). Descriptive statistics (means, standard deviations, frequencies, and percentages) were calculated. Normality was assessed by examining the standardized residuals’ histograms and normal probability (P–P) plots, which revealed a fairly normal distribution. Multicollinearity was assessed using statistics from the Variance Inflation Factor (VIF) and tolerance. As all tolerance values were above the 0.10 threshold and all VIF values were below the commonly used cutoff of 5, multicollinearity was not considered a threat to the stability of the regression model. In line with the Technology Acceptance Model (TAM), the analytical strategy was designed to examine the relationships between key constructs. Independent *t*-tests and ANOVA compare group means, and Pearson’s correlation was used to assess relationships among variables. Post hoc pairwise comparisons using Tukey’s test were conducted to identify specific group differences. Multiple linear regression analysis was conducted to identify predictors of attitudes toward AI, consistent with the TAM assumptions that perceived usefulness and perceived ease of use influence attitudes. Statistical significance was set at *p* < 0.05.

### 2.7. Ethical Considerations

Ethical Approval No obtained from the Institutional Review Board of the University of Ha’ il (Approval No. H2024-232). Participation in the study was voluntary, and no incentives were provided. The survey invitation clearly informed the students that participation was optional, and that declining to participate would have no academic or institutional consequences. The questionnaire was administered anonymously using Google Forms and no personally identifiable information was collected. All responses were kept confidential and analyzed in aggregate form for research purposes only. Electronic informed consent was obtained from all participants before they completed the survey.

## 3. Results

As shown in Table 1, the majority of participants were aged between 20 and 21 years (40.3%) with a mean ± SD of 23.56 ± 3.72, which reflects the inclusion of students across multiple academic levels, including internship-year students. More than half of the participants were female (55.6%). More than two-thirds (64.7%) were from urban areas and the majority (23.7%) were from the second level of study.

Table 2 illustrates the relationship between students’ sociodemographic characteristics, knowledge, attitude, and preparedness toward AI. Age was not significantly associated with knowledge or preparedness (*p* > 0.05) but was significantly associated with attitude (F(3, 316) = 8.74, *p* < 0.001), with students aged ≥24 years reporting higher scores. Gender was not associated with knowledge (*p* = 0.889), whereas attitude showed a marginal difference (*p* = 0.053). However, females demonstrated significantly higher preparedness than males (t(318) = −2.65, *p* = 0.008). Residence was not significantly related to knowledge, attitudes, or preparedness (*p* > 0.05). Academic year was not associated with knowledge (*p* = 0.272) but showed significant differences in attitude (F(4, 315) = 15.46, *p* < 0.001) and preparedness (F(4, 315) = 12.19, *p* < 0.001). Internship students had the highest attitude scores, whereas preparedness increased significantly from the first year to higher academic levels.

**Figure 1:**
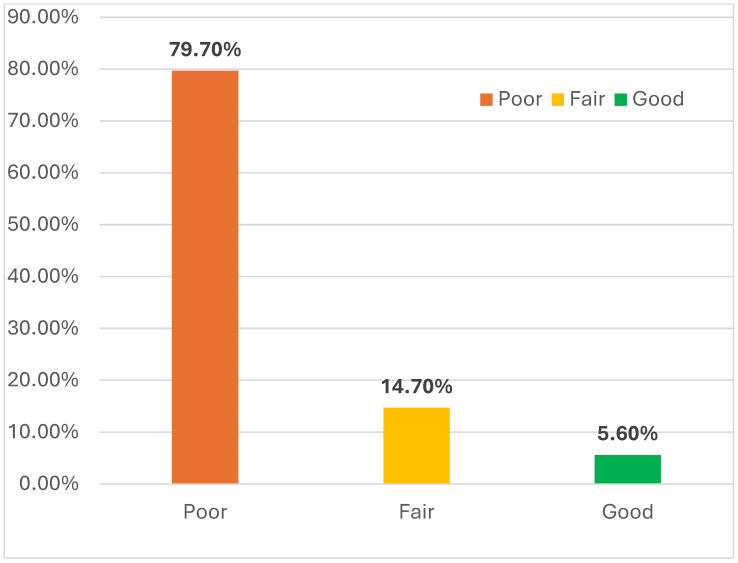
More than half of students (52.5%) had a positive attitude regarding using AI in healthcare.

As shown in Figure 1, the majority of participants (79.70%) had a poor level of knowledge, followed by fair (14.70%), while the lowest (5.60%) had a good level of knowledge.

**Figure 2:**
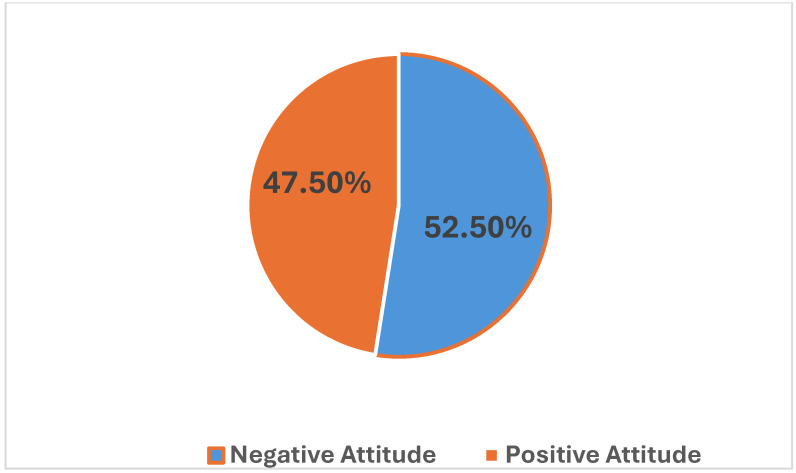
Students’ Attitudes toward Artificial Intelligence.

Figure 2 shows the distribution of students’ attitudes toward artificial intelligence. Slightly more than half of the participants reported a negative attitude (52.5%), while 47.5% demonstrated a positive attitude.

As shown in Table 3, there was a significantly weak positive correlation between knowledge and attitude (r = 0.147, *p* = 0.008), benefits (r = 0.222, *p* ≤ 0.001), and risk (r = 0.152, *p* ≤ 0.001). In relation to attitude, there was a significantly weak positive correlation between attitude and benefits (r = 0.243, *p* < 0.001), while there was a significantly weak negative correlation between attitude and barriers (r = −0.219, *p* < 0.001). In contrast, positive correlations were found between benefits and risk (r = 0.411, *p* < 0.001) and negative correlations with professional (r = −0.139, *p* = 0.013) and preparedness (r = −0.270, *p* < 0.001). There was a significant positive correlation between risk and barriers (r = 0.149, *p* = 0.008) and a negative correlation with preparedness (r = −0.143, *p* < 0.010). Finally, a significant positive correlation was found between professional skills and preparedness (r = 0.416, *p* < 0.001).

As shown in Table 4, the multiple regression model was significant (*p* < 0.001) and explained 23.1% of the variance in attitudes toward AI (R^2^ = 0.231; Adjusted R^2^ = 0.207). Fourth-year students (β = 0.170, *p* = 0.009) and internship students (β = 0.268, *p* < 0.001) showed significantly more positive attitudes toward AI than first-year students. Perceived benefits were positively associated with attitude (β = 0.171, *p* = 0.002), whereas perceived barriers were negatively associated (β = −0.143, *p* = 0.006). Age and knowledge were not significant predictors (*p* > 0.05). The second regression model examining preparedness was also significant (*p* < 0.001), explaining 30.8% of the variance (R^2^ = 0.308; Adjusted R^2^ = 0.209). Academic level emerged as a strong predictor, with second-, third-, fourth-, and internship-level students demonstrating significantly higher preparedness than first-year students (*p* < 0.001). Female students reported higher preparedness than did male students (β = 0.120, *p* = 0.011). Perceived benefits were negatively associated with preparedness (β = −0.153, *p* = 0.005), whereas perceived professional impact showed a strong positive association (β = 0.347, *p* < 0.001).

## 4. Discussion

With increasing interest in using AI in healthcare for better and more effective diagnosis, prognosis, and treatment [5], many medical and healthcare organizations emphasize the need to acquire basic knowledge of AI for healthcare workers [23,24]. As nurses constantly interact with patients and other healthcare professionals during the healthcare delivery process, they also need to be knowledgeable about the importance of AI in healthcare and prepared to integrate it [5,25,26]. Few studies have addressed the preparedness of nursing applicants and students for AI [5,9]. The findings of this study provide important insights into the existing literature regarding the knowledge, attitudes, benefits, risks, barriers, professional impact, and preparedness of nursing students toward the utilization of AI in healthcare. The results demonstrated that while the majority of nursing students demonstrated positive attitudes towards AI, their overall knowledge remained considerably low. This disparity suggests a potential gap in nursing education on AI. However, this interpretation should be considered cautiously given the limitations of the knowledge measure used in this study.

### 4.1. Knowledge and Attitudes

According to the survey, a significant majority of the included students (79.7%) had an insufficient understanding of AI, whereas only 5.6% of the students had strong knowledge of the subject. This outcome is consistent with other research indicating that despite AI’s increasing importance of AI in healthcare, nursing practitioners and students often do not sufficiently understand it [27,28]. However, the knowledge in this study was assessed using a limited number of dichotomous items without established validity in this population, which may have affected the robustness of this finding. Moreover, most studies show that nurses have insufficient awareness and experience of AI [23,28]. According to Castagno et al. (2020), 64% of nurses in Britain have no prior experience with AI and 87% are unable to distinguish between different AI concepts [28]. Although AI adoption in nursing is still emerging globally, these broader observations should not be directly generalized to the present sample, as actual exposure to AI was not measured. Despite the knowledge gap, our findings show a sense of optimism about artificial intelligence through positive attitudes. Over half of the participants (52.5%) had a favorable opinion regarding the application of AI in healthcare, suggesting that students might be more accepting of new technologies if they are provided with the necessary information and tools [5]. According to a rapid review, more than 70% of nurses believe that AI may result in a nursing revolution by improving health promotion, providing customized treatments, and automating administration and routine chores [5]. However, the relationships between attitudes and the other variables in this study were weak and should not be interpreted as strong.

The results showed that older students (≥24 years) and internships had more positive attitudes towards AI. This may be explained by the fact that being more exposed to clinical practice may result in a more favorable outlook on AI usage. However, this remains speculative, as actual exposure to AI technologies has not been assessed. Although differences in attitude were observed among students with varying levels of clinical exposure, these findings should be interpreted with caution. Due to the cross-sectional design, the results cannot establish directional or causal relationships, and the observed differences may reflect other unmeasured contextual or educational factors.

### 4.2. Perceived Benefits and Risks

Our findings demonstrate a positive correlation between knowledge level and perceived benefits (r = 0.222, *p* < 0.001). In fact, as students become more informed about AI, they are more likely to perceive its benefits and advantages in promoting healthcare delivery, such as enhancing the accuracy of diagnoses, facilitating administrative tasks, and supporting decision-making processes [29]. A study published by Said et al. found that AI Knowledge predicts people’s risk-benefit perceptions; higher AI knowledge significantly influences participants’ benefit assessment [30]. Although statistically significant, the magnitude of this association was small in our study, indicating a limited relationship. However, such interpretations remain ambitious given the modest strength of the observed association.

Nonetheless, our study also identified concerns regarding the risks associated with AI, specifically ethical issues, patient privacy, and the potential for AI to take away human jobs in healthcare in the future. These findings were corroborated by several recent studies [27,29,31]. For instance, Sit et al. (2020) found that almost half of the interviewed students believed that certain medical specialties would be replaced by AI, which would reduce the workforce in medical and nursing specialties [31].

However, an item-level analysis of specific risks was not conducted, limiting the ability to determine which concerns were most prominent among the participants.

### 4.3. Barriers to AI Utilization

One of the findings of our study was a weak negative correlation between attitudes and perceived barriers (r = −0.219, *p* < 0.001). While statistically significant, a small effect size indicated a limited association, suggesting that students who reported fewer perceived barriers tended to have more favorable attitudes toward AI. However, this relationship should be interpreted with caution because the cross-sectional design does not allow for any inferences regarding directionality or causality. It is equally plausible that students with more positive attitudes may be less likely to perceive barriers than those that directly influence attitudes.

Importantly, the study did not assess the frequency, intensity, or relative importance of specific barriers, which limits the ability to identify factors that are most salient in shaping students’ perceptions. As a result, commonly cited barriers, such as fear of job displacement, insufficient training, or concerns about reliability, cannot be confirmed as dominant within this sample. Therefore, these interpretations should be contextualized in the existing literature rather than empirically derived from the present dataset. According to Kwak et al., nurses and nursing students are concerned about job loss and the possibility of full automation, which creates resistance to AI adoption [32]. With this in mind, anxiety and negative perceptions surrounding AI stem from insufficient education and sensitization, leaving many professionals ill-prepared and closed-minded towards AI technologies [28,32]. From another perspective, there are major concerns about the possibility of errors, unexpected outcomes, and trustworthiness of AI that further complicate its incorporation into clinical practice because healthcare workers require a high level of accuracy and reliability to ensure patient safety [28,32].

The findings of this study can be analyzed using the Technology Acceptance Model (TAM). Consistent with the TAM, perceived advantages (as a measure of perceived utility) were favorably connected with attitudes toward AI, whereas perceived barriers (representing perceived ease of use) were negatively related. However, the modest correlations revealed indicate that while TAM is a valuable explanatory framework, additional environmental and educational elements may influence students’ acceptance of AI [7]. This emphasizes the necessity to look beyond basic TAM characteristics when analyzing technology adoption in complicated educational and therapeutic settings.

### 4.4. Professional Impact and Preparedness

While the students expressed a positive attitude toward AI, their preparedness to integrate it into professional practice was limited. A weak correlation was found between knowledge and preparedness (r = 0.005), indicating a lack of meaningful relationships. Halat et al. showed that students’ readiness and preparedness to use AI had a significant correlation with AI perceptions and perceived level of AI knowledge [33].

Unfortunately, this preparedness construct was not adequately captured in our study. However, a recently published study by Testa et al. suggested that preparedness is not determined by theoretical knowledge alone and highlighted the importance of experiential learning rather than conceptual understanding [34].

Regarding professional impact, while strong conclusions cannot be drawn from the present data, integrating AI-related competencies remains a relevant consideration [35]. AI may benefit nurses and nursing students in numerous ways, as it has the potential to improve nursing care in a clinical setting by facilitating decision-making and promoting nursing care plans during patient treatment [34,35]. Based on research, curriculum development should be prioritized and focused on a competency-based curriculum, spiral learning structures, multidisciplinary strategies, and experiential engagement to equip students with the changing landscape of healthcare technology by integrating AI across courses, from basic to clinical, and incorporating ethical considerations [13,14,34,36].

#### Implications for Nursing Education

Beyond documenting knowledge and attitudes, our findings have significant implications for health care education and workforce development. Identifying specific areas of lack of knowledge can help build focused educational interventions and curricular modifications that improve students’ readiness to face health care concerns. As a result, these findings will help improve the preparation of future healthcare workers, allowing them to provide better patient care.

### 4.5. Limitations

Although our study provided valuable insights into nursing students’ knowledge, attitudes, perceived benefits, risks, barriers, professional impact, and preparedness regarding AI in healthcare, several limitations must be acknowledged. However, this study had several limitations. First, our cross-sectional approach may not fully reflect changing and evolving attitudes toward AI technologies, especially in the context of rapidly shifting media narratives. Moreover, our study was based on a self-administered questionnaire, which may have introduced potential bias, including recall bias and social desirability bias, for which the participants may have given answers that they thought were acceptable rather than their real attitudes or knowledge. Additionally, the survey was administered online, and there is also the possibility of self-selection bias, as students who are more comfortable with digital technologies or more interested in the topic may have been more likely to participate. Third, participants were recruited using convenience sampling from a single academic institution, which may have introduced sampling bias. Therefore, the study sample may not be fully representative of nursing students from other universities, regions, or educational contexts. Consequently, the generalizability of our findings should be cautiously interpreted. Future studies should consider using multicenter sampling strategies and probability-based sampling methods to improve representativeness and external validity. In addition, the unequal distribution of participants across academic years may have influenced the study findings, particularly since students with more advanced clinical training may have different levels of knowledge or attitudes than junior students. We also did not conduct item-level analyses for risks and barriers, and actual exposure to AI technologies was not measured, thus limiting the interpretation of the associations with attitudes and preparedness. Additionally, the use of multiple statistical tests may increase the risk of Type I errors. Although no formal correction (e.g., Bonferroni adjustment) was applied, the results were interpreted cautiously with attention to effect sizes and theoretical consistency. Finally, the use of dichotomous items may reduce the sensitivity and limit the accurate assessment of AI knowledge.

## 5. Conclusions

This study demonstrated a clear gap between nursing students’ positive attitudes toward artificial intelligence (AI) and their limited knowledge and preparedness for clinical application. Although more than half the students expressed favorable perceptions of AI, the majority showed poor knowledge and low readiness to use AI in healthcare settings. Older students and those in their internship years exhibited more positive attitudes, indicating that clinical exposure may enhance the acceptance of AI. The significant associations between knowledge, perceived benefits, risks, barriers, and preparedness highlight the complex factors that influence students’ readiness for AI integration. These findings highlight the need for targeted education strategies. For curriculum designers, integrating structured educational modules, case-based learning, and simulation-based training into nursing curricula may help strengthen students’ knowledge and practical competencies. Clinical educators can support this effort by incorporating experiential learning opportunities and clinical discussions to reinforce theoretical knowledge in real-world practice. At the policy level, academic institutions and healthcare authorities should consider developing standardized educational guidelines and continuing training initiatives to ensure that nursing students and future healthcare professionals are adequately prepared to address this issue. These strategies may contribute to improving competency development and ultimately enhance the quality of healthcare delivery. However, these results should be interpreted with caution given the cross-sectional design, single-institution setting, convenience sampling, and reliance on self-reported measures.

## Figures and Tables

**Table 1:** Socio-demographic characteristics of the students (N = 320).

Demographic Characteristics		n	%
Age in years	18–<20	82	25.6
	20–<22	129	40.3
	22–<24	69	21.6
	≥24	40	12.5
	Mean ± SD	23.56 ± 3.72	
Gender	Male	142	44.4
	Female	178	55.6
Residence	Urban	207	64.7
	Rural	113	35.3
Academic year	First	61	19.1
	Second	76	23.7
	Third	69	21.6
	Fourth	60	18.7
	Internship year	54	16.9

**Table 2:** The relationship between students’ sociodemographic and knowledge, attitude, and preparedness toward AI.

Variables		Knowledge			Attitude			Preparedness		
		Mean ± SD	t/f (df)	(*p*-Value)	Mean ± SD	t/f (df)	(*p*-Value)	Mean ± SD	t/f (df)	(*p*-Value)
Age										
	18–<20 ^a^	6.47 ± 2.75	0.98 (316)	0.400	28.86 ± 4.63	8.74 (316)	<0.001	7.56 ± 2.52	2.28 (3)	0.079
	20–<22 ^b^	6.21 ± 2.58			28.89 ± 6.72		* d > a, * d > b, * d > c	7.79 ± 2.87		
	22–<24 ^c^	5.82 ± 2.12			29.14 ± 4.76			7.44 ± 2.09		
	≥24 ^d^	6.42 ± 1.76			33.72 ± 3.86			6.60 ± 2.10		
Gender										
	Male	6.24 ± 2.18	0.14 (318)	0.889	28.87 ± 4.85	−1.88 (318)	0.053	7.09 ± 2.11	−2.65 (318)	0.008
	Female	6.20 ± 2.65			30.08 ± 6.29			7.84 ± 2.83		
Residence										
	Urban	6.14 ± 2.51	−0.83 (318)	0.392	29.23 ± 5.41	−1.31 (318)	0.190	7.54 ± 2.58	0.31 (318)	0.753
	Rural	6.38 ± 2.33			30.11 ± 6.24			7.45 ± 2.52		
Academic year										
	First ^a^	6.83 ± 2.48	1.29 (315)	0.272	29.14 ± 4.31	15.46 (315)	<0.001	5.73 ± 2.48	12.19 (4)	<0.001
	Second ^b^	5.93 ± 2.20			26.65 ± 5.67		* d > b, * e > a, * e > b, * e > c, * e > d,	7.93 ± 2.40		* b > a, * c > a, * d > a, * e > a
	Third ^c^	6.11 ± 2.68			28.68 ± 5.74			8.55 ± 2.68		
	Fourth ^d^	6.21 ± 2.51			30.91 ± 5.68			7.50 ± 2.48		
	Internship ^e^	6.09 ± 2.32			33.64 ± 4.46			7.61 ± 1.68		

T = independent sample *t*-test; f = ANOVA test; a, b, c, d, and e indicate that each group was compared using Tukey’s post hoc test; * = significant post hoc test. Effect sizes were reported as Cohen’s d for two-group comparisons and η^2^ for ANOVA tests to aid in the interpretation of practical significance.

**Table 3:** Correlation between study variables.

Variables		Knowledge	Attitude	Benefits	Risk	Barrier	Professional	Preparedness
Knowledge	Pearson’s r	1	0.147	0.222	0.152	−0.061	0.029	0.005
	*p*-value		0.008	<0.001	0.006	0.278	0.599	0.934
Attitude	Pearson’s r		1	0.243	0.025	−0.219	0.092	0.058
	*p*-value			<0.001	0.658	<0.001	0.101	0.303
Benefits	Pearson’s r			1	0.411	−0.092	−0.139	−0.270
	*p*-value				<0.001	0.100	0.013	<0.001
Risk	Pearson’s r				1	0.149	−0.049	−0.143
	*p*-value					0.008	0.384	0.010
Barrier	Pearson’s r					1	−0.024	−0.030
	*p*-value						0.663	0.594
Professional	Pearson’s r						1	0.416
	*p*-value							<0.001

Correlation is significant at the 0.01 level (2-tailed). Correlation is significant at the 0.05 level (2-tailed).

**Table 4:** Multiple regression of Factors affecting nursing students’ attitude and preparedness toward AI.

Variables	Categories	Attitude *						Preparedness **					
		B	SE	β	t	95% CI	*p*-Value	B	SE	β	t	95% CI	*p*-Value
Age													
	18–<20	Reference						NA					
	20–<22	0.41	0.74	0.03	0.55	−1.04–1.86	0.579						
	22–<24	0.20	0.88	0.01	0.22	−1.53–1.93	0.821						
	≥24	1.77	1.09	0.10	1.61	−0.32–3.92	0.106						
Gender													
	Male	NA											
	Female							0.62	0.24	0.12	2.55	0.14–1.10	0.011
Level													
	First	Reference											
	Second	−1.25	0.92	−0.09	−1.36	−3.07–0.56	0.174	1.75	0.38	0.29	4.54	0.99–2.50	<0.001
	Third	0.24	0.93	0.01	0.26	−1.60–2.08	0.795	2.01	0.39	0.32	5.05	1.23–2.79	<0.001
	Fourth	2.49	0.94	0.17	2.62	0.62–4.36	0.009	1.40	0.40	0.21	3.46	0.60–2.19	<0.001
	Internship	4.09	1.03	0.26	3.95	2.05–6.12	0.000	1.52	0.40	0.22	3.76	0.72–2.32	<0.001
Knowledge		0.08	0.12	0.03	0.74	−0.14–0.32	0.456	NA					
Benefits		0.21	0.06	0.17	3.16	0.08–0.35	0.002	−0.087	0.03	−0.153	−2.847		0.005
Risk		NA						−0.01	0.06	−0.01	−0.18		0.855
Barrier		−0.45	0.16	−0.14	−2.79	−0.77–−0.13	0.006	NA					
Professional		NA						0.19	0.02	0.35	7.34	0.14–0.24	<0.001

* R2 = 0.231; Adjusted R2 = 0.207; *p* < 0.001; ** R2 = 0.308; Adjusted R2 = 0.209; *p* < 0.001; S. E, Std. Error (CI) and confidence interval.

## Data Availability

The data presented in this study are available upon request from the corresponding authors.

## Supplementary Materials

The following supporting information can be downloaded at: https://www.mdpi.com/article/10.3390/nursrep16050154/s1, STROBE Statement.

## Public Involvement Statement

No public involvement in any aspect of this research.

## Guidelines and Standards Statement

This manuscript was prepared in accordance with the STROBE (Strengthening the Reporting of Observational Studies in Epidemiology) guidelines for cross-sectional studies [37] (see Supplementary Material).

## Abbreviations

The following abbreviations are used in this manuscript:

AI	Artificial Intelligence
SD	Standard Deviation
